# Supplementary material for: A Promising Future for Stem-Cell-Based Therapies in Muscular Dystrophies—In Vitro and In Vivo Treatments to Boost Cellular Engraftment
Source: Int J Mol Sci. 2019 Oct 31;20(21):5433. doi: 10.3390/ijms20215433 (PMC6861917; doi:10.3390/ijms20215433)
Supplement: Supplementary file 1 [file ijms-20-05433-s001.pdf]

Table S1.Summary of muscular dystrophies, pathology and treatments.

Type of dystrophy	Mutation and/or related genes	Affected Protein(s)	Features	Treatments/Therapies	Reference
**Myotonic muscular dystrophy (MMD)**	Expansion of a trinucleotide (CTG) repeat on chromosome 19q13.3 for type 1 and tetranucleotide (CCTG) repeats on chromosome 3q21.3 for type 2 disease.	Myotonic dystrophy protein kinase	A dominant disorder associated with clinical myotonia, progressive muscular weakness, and extra muscular manifestations such as cardiac arrhythmia and endocrine dysfunction.	No treatments are currently available that fundamentally alter the course of MMD1 or MMD2. The management of MMD patients is based on genetic counseling and the preservation of social independence and cardiopulmonary complications by providing symptomatic treatment for myotonia, hypersomnolence, and pain. Moreover, some experimental therapeutic approaches are under investigation, as antisense oligonucleotides (ASOs), gene therapy vectors, and small molecules.	(9, 15)
**Facioscapulohumeral dystrophy (FSHD)**	Deletions within the D4Z4 repeat region located on chromosome 4q35 for type 1; mutations of SMCHD1 on chromosome 18p11 in association with a permission chromosome 4 allele account for the majority of type 2 disease. The epigenetic derepression of the	a toxic-gain-of-function DUX4 protein'	Facioscapulohumeral muscular dystrophy has a characteristic descending pattern of weakness, first affecting the face and shoulder followed by the distal lower extremity and the proximal hip girdle muscles, but many variations in the presentation can occur.	There are no registered therapies approved for FSHD. Although many drugs have been tested in clinical trials (prednisone diltiazem, albuterol, and a myostatin inhibitor), none showed a clear benefit. Stretching and range of motion exercises are also recommended	(43, 44, 47)

	anormally silenced gene DUX4 (double homeobox 4).				
**Duchenne muscular dystrophy (DMD) and Becker muscular dystrophy (BMD)**	X-linked recessive allelic disorders caused by mutations in the dystrophin gene	Dystrophin	The absence of dystrophin leads to progressive muscle necrosis, loss of independent ambulation by early adolescence, cardiomyopathy, respiratory insufficiency, and premature death in DMD individuals. BMD is caused by mutations in the same gene, leading to the production of an altered protein and the late onset of symptoms. BMD patients also have skeletal muscle weakness, but cardiac dysfunction in more pronounced	So far, therapy aimed at a complete recovery has not been developed for these diseases. The most appropriate therapy would be complementation or restoration of dystrophin expression, such as gene therapy using viral vectors, read through therapy, or exon skipping therapy.	(56, 59, 60)
**Emery- Dreifuss Muscular Dystrophy (EDMD)**	Associated with mutations in the emerin gene (*EMD*) located in the X chromosome and other genes, such as FHL1, LMNA, SYNE1, SYNE2	Emerin, LaminA/C, Nesprin	This disorder is characterized by childhood onset of early contractures, humeroperoneal muscle atrophy, and cardiac conduction abnormalities. Weakness is slowly progressive, but there is a broad spectrum of clinical severity. Patients and carriers are at risk of sudden death	There is no specific treatment. All patients should have a detailed cardiac investigation and regular follow-up by cardiologist since sudden death can occur in these patients. Early detection of arrhythmias can be lifesaving by pacemaker or defibrillator implantation. Specific therapeutic approaches have been developed in animal models	(32, 25, 26)
**Limb-girdle muscular dystrophy (LGMD)**	Multiple genes involved. Type 1 (dominant) or type 2 (recessive); caused	Sarcoglycan, Dystroglycan,	Ambulation typically achieved, sometimes lost at near adulthood or middle age; intellectual	Treatment in LGMDs has mostly been symptomatic so far, with a focus on treating respiratory and	(27, 39)

	by mutations involving extracellular matrix or external membrane proteins, enzymes or proteins with putative enzymatic function, sarcolemma-associated proteins, nuclear membrane proteins, sarcomeric proteins, and other as-yet unspecified disorders.	Telethonin, Titin, and so forth	disabilities; variable cardiomyopathy (common in sarcoglycan deficiency, dystroglycanopathy)	cardiac deficiency, contracture prophylaxis, and surgery for scoliosis and short tendons. Other ongoing treatments in LGMD patients include a clinical trial of coenzyme Q10 and lisinopril in LGMD 2C–2F.	
**Oculopharyngeal muscular dystrophy (OPMD)**	Repeat expansion in the PABPN1 gene that results in an N-terminal expanded polyalanine tract in polyA-binding protein nuclear 1 (PABPN1)	polyadenylate binding protein (PABPN1)	OPMD is mainly characterized by progressive eyelid drooping, swallowing difficulties (as the pharyngeal muscles are mostly affected) and proximal limb weakness.	No cure is currently available to arrest the disease. Surgical pharyngeal myotomy is the only treatment available to improve swallowing in these patients. Pharmacological therapies, currently under pre-clinical investigation, include antiprion drugs like 6-aminophenanthridine and guanabenz, and targeting the expPABPN1 using intracellular antibodies. However, none of these strategies directly correct the genetic defect of OPMD patients.	(21-23)
**Congenital muscular dystrophy (CMD)**	There are more than 13 genes associated with CMD. Among them are: **1.**CMD with partial merosin deficiency	Multiple	Hypotonia, muscle weakness, and reduced deep tendon reflexes, with or without joint contractures; microcephaly, eye anomalies, cerebral	There is no pharmacological treatment for the CMDs. At present, the treatment is to ameliorate the course of the disease and prevent or treat	(50, 38)

	(MDC1B with locus in 1q42); **2.**LARGE related CMD (MDC1D—gene *LARGE*); **3.** Fukuyama CMD, *FCMD* gene, protein Fukutin; **4.** Muscle-Eye-Brain (MEB) genes *POMGnTI, FKRP, ISPD, TMEM5,* Fukutin); **5.** Walker-Warburg syndrome (WWS), genes *POMTI, POMT2; FKRP, ISPD, CTDC2, TMEM5, POMGnTI, B3GALNT2, GMPPB, B3GNT1, SGK196,* Fukutin); **6.** CMD/LGMD with Mental Retardation (genes *FKRP, POMTI, POMT2, ISPD, GMPPB*); **7.** CMD/LGMD without mental retardation including MDC1C (genes *FKRP, ISPD, GMPPB,* Fukutin).		malformation, joint laxity, muscle atrophy, or hypertrophy	pulmonary and cardiac impairment. Supportive treatment with non-invasive respiratory support in case of respiratory distress, correction of gastroesophageal reflux, support in cardiac failure, treatment of respiratory infections, and nutritional therapy are important.	
